# An Evolutionary-Focused Review of the *Holosporales* (*Alphaproteobacteria*): Diversity, Host Interactions, and Taxonomic Re-ranking as *Holosporineae* Subord. Nov

**DOI:** 10.1007/s00248-025-02509-0

**Published:** 2025-03-14

**Authors:** Michele Castelli, Giulio Petroni

**Affiliations:** 1 Department of Biology and Biotechnology, University of Pavia, Pavia, Italy; 2 Department of Biology, University of Pisa, Pisa, Italy

**Keywords:** *Paramecium*, *Acanthamoeba*, R-body, Bacterial endosymbionts, Protists, *Rickettsiales*, *Holospora*, *Caedibacter*/*Caedimonas*, Intranuclear bacteria, Killer trait, Holosporaceae, Holosporales, Holosporineae

## Abstract

The order *Holosporales* is a broad and ancient lineage of bacteria obligatorily associated with eukaryotic hosts, mostly protists. Significantly, this is similar to other evolutionary distinct bacterial lineages (e.g. *Rickettsiales* and *Chlamydiae*). Here, we provide a detailed and comprehensive account on the current knowledge on the *Holosporales*. First, acknowledging the up-to-date phylogenetic reconstructions and recent nomenclatural proposals, we reevaluate their taxonomy, thus re-ranking them as a suborder, i.e. *Holosporineae*, within the order *Rhodospirillales*. Then, we examine the phylogenetic diversity of the *Holosporineae*, presenting the 20 described genera and many yet undescribed sub-lineages, as well as the variety of the respective environments of provenance and hosts, which belong to several different eukaryotic supergroups. Noteworthy representatives of the *Holosporineae* are the infectious intranuclear *Holospora*, the host manipulator ‘*Caedimonas*’, and the farmed shrimp pathogen ‘*Candidatus* Hepatobacter’. Next, we put these bacteria in the broad context of the whole *Holosporineae*, by comparing with the available data on the least studied representatives, including genome sequences. Accordingly, we reason on the most probable evolutionary trajectories for host interactions, host specificity, and emergence of potential pathogens in aquaculture and possibly humans, as well as on future research directions to investigate those many open points on the *Holosporineae*.

## Overview and Purposes

The *Holosporales* are an alphaproteobacterial order that was taxonomically described in the last decade [1], but research on their members has a long-lasting tradition in microbiology, in particular on two noteworthy representatives, namely the eponym *Holospora* and ‘*Caedimonas*’, both living intracellularly in ciliate protists. *Holospora* spp. are highly infectious bacteria inhabiting the nucleus of *Paramecium* hosts [2, 3], and their description dates back to the seminal work by Mardukhey Wolf-Vladimir Hafkin in the late nineteenth century [4]. ‘*Caedimonas varicaedens*’ confers its *Paramecium* hosts the so-called ‘killed trait’, similarly to the gammaproteobacterium *Caedibacter taeniospiralis*, and was indeed ascribed to the *Caedibacter* genus before molecular phylogenies showed their unrelatedness [5–7]. Long before understanding that the causative agents were actually bacteria, the killer trait was identified as early as 1938 in the pioneering investigations by Tracy Sonneborn [8].

Over the last three decades, extensive molecular surveys of host-associated bacteria showed that *Holospora* and ‘*Caedimonas*’ are phylogenetically close and form a conspicuous lineage together with several other bacteria [5, 9–12]. Currently, this lineage is formally described at the order rank (*Holosporales*) (https://lpsn.dsmz.de/order/holosporales) following Szokoli and co-authors [1]. As motivated in details below, here we will propose to rank it as a suborder (i.e. *Holosporineae*). Until the dedicated section, here, we will use taxonomic terms according to Szokoli and co-authors unless otherwise specified.

The *Holosporales* are likely very ancient (over 1 billion years) [13], and their extant members characterised so far are solely host-associated, with the vast majority of such hosts being unicellular eukaryotes, i.e. protists [14]. The potential host association status is unknown for additional uncharacterised members of the *Holosporales*, known only by their DNA sequences obtained in environmental screening and/or metagenomic studies (e.g. [15–27]), in several cases with more or less strong indications of association to eukaryotic hosts, e.g. [28–37]. Accordingly, it seems probable that the association with protists (and other eukaryotes) dates back to the last common ancestor of the *Holosporales*.

Their evolutionary antiquity, diversity breadth, and ancient host-association make the *Holosporales* a noteworthy subject of study and research, bringing them together with other well-known bacterial lineages with ancient host-association, such as *Rickettsiales*, *Chlamydiae*, and *Legionellales* [38–40]. The common features of those lineages (including the *Holosporales*) led some authors to define them as ‘professional symbionts’, underlining their ability to adapt to varied hosts and in some way presumably take control of the interactions [41, 42]. Therefore, the study of *Holosporales* is particularly relevant for comparisons with other professional symbionts, in order to highlight evolutionary convergent or lineage-specific traits. For example, the evolutionary origin and possible conservation of functional features of the killer trait of ‘*Caedimonas*’ has a broad significance, as it may be seen as a host addictive manipulation phenomenon [43]. Last but not least, differently from other professional symbiont lineages [44–46], the *Holosporales* do not encompass renowned human pathogens, but they do include the members of the genus ‘*Candidatus* Hepatobacter’ [10, 47, 48], which affect farmed shrimps and other crustaceans, causing necrotising hepatopancreatitis (NHP), a disease recognised by the World Organization for Animal Health [49].

The research interest on the *Holosporales* is shown by numerous research papers produced in recent years (e.g. [14, 34, 50–58]). However, to our best knowledge, there is not yet a dedicated review piece focused on the *Holosporales* as a whole and on their evolutionary and functional features, possibly due to their relatively recent recognition as a high-rank and independent lineage [1, 59]. Here, we aim to fill this gap, dealing with the following subjects:

- Taxonomical overview and proposed revisions
- Diversity of representatives and the respective hosts
- Evolution of the lineage with a focus on the interactions with the hosts, informed by genomics

## The Taxonomy of the *Holosporales*: Historical Overview and Proposed Revisions

The phylogenetic reconstructions of the *Holosporales* and the consequent taxonomic views have changed multiple times across recent years. Although not all taxonomic proposals were formally validated, this inevitably led to some incongruence among different studies published within short timings from one to another, each referring to a different taxonomical version, with a substantial risk to mislead non-specialists. Here, we will review in chronological order the main changes in the taxonomy of *Holosporales*, showing in particular the current ambiguities and the reasons that led us to propose here a novel revision that, in our view, should settle the issue for long.

The taxonomic affiliation of *Holospora* was initially uncertain [60]. Early molecular phylogenies based on 16S rRNA gene sequences indicated its relatedness to the order *Rickettsiales* [61], forming a distinct early-diverging lineage together with other bacteria associated with ciliates and other protists (including ‘*Caedimonas*’) [5, 9, 11, 62, 63], as well as with metazoans [10]. Accordingly, Görtz and Schmidt created a novel family for these bacteria within the *Rickettsiales*, namely the *Holosporaceae* [64], including also few other symbionts of ciliates that were not yet characterised molecularly. Curiously, molecular phylogenies later disproved the affiliation to the *Holosporaceae* of some of the latter, namely *Lyticum* and *Pseudolyticum*, both actually belonging to the ‘*Candidatus* Midichloriaceae’ (*Rickettsiales*) [65, 66], while molecular data are still lacking for others (*Pseudocaedibacter* and *Tectibacter*) [67, 68], making their actual affiliation uncertain to date.

Later on, based on the strong synapomorphies in the morphology and life cycle among *Holospora* and *Holospora*-like bacteria (HLB) [3, 4], Boscaro and colleagues informally proposed to restrict the *Holosporaceae* only to those bacteria, while treating all other *Holosporaceae *sensu Görtz and Schmidt as *incertae sedis* within the *Rickettsiales* [69].

In the same years, the phylogeny and taxonomy of these bacteria were influenced by multiple studies. On one hand, multiple other members of the *Holosporaceae *sensu Görtz and Schmidt were identified, e.g. [29, 70–75], with Hess and colleagues formally proposing to elevate at the family rank a sublineage including multiple symbionts of amoebas, namely the ‘*Candidatus* Paracaedibacteraceae’ [75]. Moreover, seminal phylogenetic studies with extended molecular markers [76–78] cast significant doubt on the actual affiliation of the *Holosporaceae *sensu Görtz and Schmidt to the *Rickettsiales*, which had relied on the 16S rRNA gene. Specifically, Ferla and colleagues made a first informal proposal to elevate them at the order rank [77].

Accounting for the above findings, Szokoli and colleagues formally re-organised the taxonomy and systematics of these bacteria [1], re-describing the *Holosporales* as an order, with four families, which is the taxonomy formally accepted to date (https://lpsn.dsmz.de/order/holosporales). These families are the ‘*Candidatus* Paracaedibacteraceae’ sensu Hess and co-authors, the *Caedibacter*/*Nucleicultrix* clade (soon after described as ‘*Caedimonadaceae*’ by Schrallhammer and colleagues, jointly with the description of ‘*Caedimonas*’ as a distinct genus from *Caedibacter* [7]), the *Holosporaceae* (revised in order to include HLB and their close relatives, thus narrower than sensu Görtz and Schmidt, but broader than the proposal by Boscaro and colleagues), and the newly described ‘*Candidatus* Hepatincolaceae’. The latter encompass mostly arthropod-associated bacteria [1, 79, 80] but have been recently shown to be phylogenetically unrelated to the *Holosporales* [81]. Below, we will propose a systematic revision for them as well.

Soon afterwards, the phylogenetic placement of the *Holosporales* was reassessed by Muñoz-Gómez and co-authors, who provided substantial evidence that the placement within *Rickettsiales* was an artefact due to compositional biases and that the *Holosporales* are actually related to, and possibly nested within, another broad and ancient alphaproteobacterial order, namely the *Rhodospirillales* [59]. Accordingly, they proposed to down-rank the *Holosporales* at the family level (to be named once again *Holosporaceae*) within the *Rhodospirillales* and to down-rank their families recognised at the time as subfamilies (i.e. *Holosporodeae*, ‘*Candidatus* Paracaedibacteriodeae’, and ‘*Candidatus* Hepatincolodeae’).

While the taxonomic revision proposed by Muñoz-Gómez and co-authors was not formally validated, it was adopted by a number of successive studies, e.g. [54, 82], in parallel to the version by Szokoli and co-authors in others, e.g. [55–57]. The presence of these two alternative versions is quite unfortunate, especially when considering that both use the term *Holosporaceae* with different meanings.

This taxonomic scenario is further complicated by the revisions recently proposed by Chuvochina and co-authors based on the Genome Taxonomy Database (GTDB) [83]. These consist in subdividing the members of the *Holosporales* into three orders roughly corresponding to the three families accounted by Szokoli and co-authors, namely *Holosporales*, ‘*Candidatus* Paracaedibacterales’, and ‘*Caedimonadales*’, and in proposing two additional families, namely the ‘*Candidatus* Hepatobacteraceae’ (*Holosporales*) and ‘*Candidatus* Nucleicultricaceae’ (‘*Caedimonadales*’). Those novel proposals are currently treated as non-standing heterotypic synonyms (https://lpsn.dsmz.de/). The discrepancies with respect to other classifications are partly due to the fact that the inference of the underlying phylogenetic backbone did not counteract the known artefacts evidenced by Muñoz-Gómez and co-authors, thus resulting in incorrect placing and splitting of the *Holosporales* (sensu Szokoli et al.). However, they are also due to different and more stringent thresholds used to delineate taxonomic ranks. It seems worth to consider that, given such thresholds, Chuvochina and co-authors also proposed to elevate at the order rank several lineages that are affiliated to the *Rhodospirillales* according to the currently validated taxonomy [84], namely *Acetobacterales*, *Azospirillales*, *Geminicoccales*, *Oceanibaculales*, *Reyranellales*, *Thalassobaculales*, and *Tistrellales* [83].

We believe that the current taxonomic ambiguities on the *Holosporales* should be settled by an evolutionarily and biologically meaningful proposal that meets the following criteria: (i) consistency with most up-to-date phylogenetic reconstructions, (ii) consistency and compatibility with most up-to-date (and ideally also foreseen) taxonomic classification schemes of *Alphaproteobacteria*, (iii) compliance to the formal standards for validation. The most credible phylogenetic backbone for the *Holosporales* is the one obtained by Muñoz-Gómez and co-authors, namely phylogenetically nested within the *Rhodospirillales *sensu Hördt et al., as confirmed by successive studies [40, 85]. While the taxonomic proposal by Muñoz-Gómez and co-authors accounts for this phylogeny, it also implies the down-ranking of the families of *Holosporales* as subfamilies, which does not account for the great phylogenetic diversity of those, the same that led Chuvochina and co-authors to rank them as separate orders. We believe that the most suitable solution is intermediate between those extremes. Accordingly, we formally propose to down-rank the *Holosporales *sensu Szokoli et al. (corresponding to the *Holosporaceae *sensu Gortz and Schmidt and sensu Muñoz-Gómez et al.) as a suborder within the *Rhodospirillales *sensu Hördt et al., namely *Holosporineae*. Accordingly, the rank and composition of the included families should be kept unchanged as per Szokoli et al. This novel taxonomic proposal has the additional advantage that it would suit well to potential future revisions of high-order taxonomy of the *Alphaproteobacteria*, including subdividing the *Rhodospirillales* into multiple orders, should a ‘splitter’ view similar to the one by Chuvochina and co-authors eventually prevail. Indeed, in such a case, the *Holosporineae* could be meaningfully re-elevated at the order rank, but, differently from other previous proposals, without the need of further changes in their inner subdivisions, thus simplifying revisions and favouring nomenclatural consistency over time. From now on, here, we will refer to this lineage as *Holosporineae*. Moreover, accounting for the most recent phylogenetic evidence of an independent branching of the ‘*Candidatus* Hepatincolaceae’ and *Holosporineae* within the *Rhodospirillales* [81], here, we formally propose to move this family out of the *Holosporineae*. Those taxonomic revisions are presented at the end of the text.

## Diversity of the *Holosporineae* and Their Hosts

All the characterised *Holosporineae* (i.e. *Holosporales *sensu Szokoli et al.) were consistently retrieved as intracellular bacteria in multiple eukaryotic hosts, predominantly protists [1, 3, 7, 14, 41, 54, 57, 59, 75]. Therefore, here, we will assume that all the *Holosporineae* are host-associated and will treat those representatives sequenced in environmental screening studies as putatively associated with unknown hosts. Accordingly, their environmental provenance would actually reflect the one of the respective hosts, notwithstanding the possibility of detecting temporarily free transmission forms of the bacteria, such as the infectious forms of *Holospora* [2, 3]. Among the *Holosporineae* putatively associated with Metazoa, only the members of the ‘*Candidatus* Hepatobacter’ genus were investigated in detail and confirmed as intracellular in the host cells [10, 47, 48]. On the other hand, all the others were sequenced from samples coming from the host gut [28, 32, 36, 86–90] or skin [91–94]. In those cases, a hypothetical intracellular association with the target host is still to be verified, while an alternative association with microbial eukaryotes, possibly ingested by the animal or part of its stable gut community, seems also plausible [95].

**Fig. 1:**
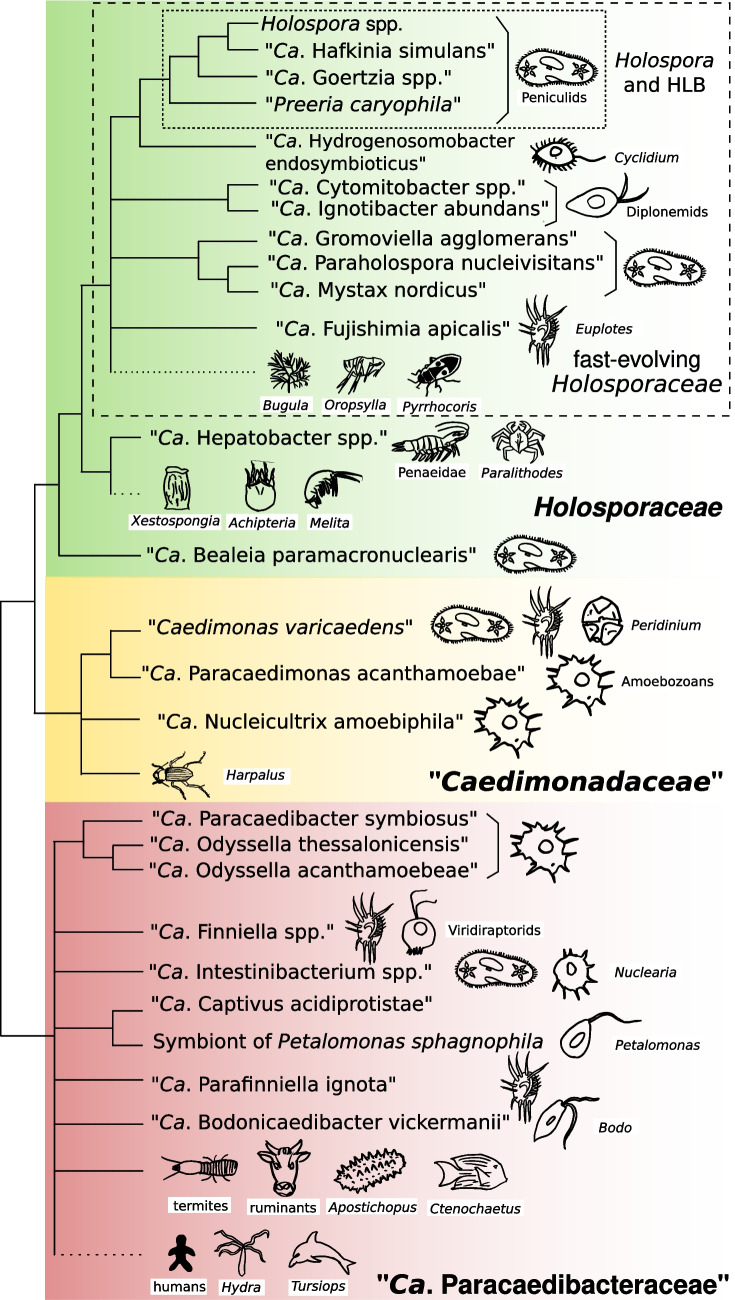
Cladogram showing the phylogenetic relationships among the *Holosporineae*, based on a combination of published reference studies, with inconsistent/unresolved relationships represented as polytomies [1, 14, 54, 57, 58, 75]. For presentation purposes, the branches leading to the ‘fast-evolving *Holosporaceae*’ are shown longer than the others. For each organism/clade, the most typical hosts are shown on the right-hand side, with name labels on the first occurrence of each host (see main text for further details). Branches without text labels indicate unnamed bacteria found through environmental screening studies in association with the shown putative hosts. Dotted branches indicate the grouping, for space constraints, of organisms that descend from the previous node in the tree, but with unconfirmed reciprocal phylogenetic relationships. The three families of the *Holosporineae* are shown by coloured backgrounds, and two subgroups of the *Holosporaceae* are encircled by dashed rectangular shapes. “*Ca*.” is an abbreviation for ‘*Candidatus*’, while ‘HLB’ for ‘*Holospora*-like bacteria’

In this section, we will review the phylogenetic diversity of the *Holosporineae*, with a focus on experimentally characterised and taxonomically described species over specimens known only from metagenomic screenings. The *Holosporineae* encompass three families, namely *Holosporaceae*, ‘*Caedimonadaceae*’, and ‘*Candidatus* Paracaedibacteraceae’ (Fig. 1). Most phylogenetic inference studies, both on the 16S rRNA gene or by phylogenomics, indicate that *Holosporaceae* and ‘*Caedimonadaceae*’ are sister groups [1, 14, 50, 54, 58, 69, 74, 75, 78, 95].

The *Holosporaceae* are mostly associated with ciliates and other protists [96] but also with arthropods, and encompass 13 described genera (Table 1). Among them, the monophyletic lineage made up of *Holospora* and the HLB (three other described genera, namely ‘*Candidatus* Goertzia’, ‘*Preeria*’, and ‘*Candidatus* Hafkinia’) is prominent (highlighted in Fig. 1) [3, 69, 97, 98]. These bacteria are associated with ciliates, prevalently of the genus *Paramecium*, and are characterised by distinctive apomorphies, including morpho-functional differentiations linked with an infectious lifecycle that involves invasion of the host nuclei [2]. Such peculiarities allowed Hafkin to notice these bacteria for the first time over 100 years ago [4] and consented a reliable assignment of new specimens to this lineage based on microscopy observations [60, 68]. Eventually, molecular data revealed the phylogenetic breadth of this genus, with five species accordingly recognised to date, namely *H. obtusa*, *H. undulata*, *H. curviuscula*, *H. acuminata*, and ‘*Candidatus* Holospora parva’, hosted by *Paramecium caudatum*, *P. bursaria*, *P. aurelia*, and *P. chlorelligerum* (Table 1) [61, 69, 99–101]. Molecular phylogenies are not always fully consistent with morphology, leading to a recent revision of *H. undulata* to encompass also the former species *H. elegans* and *H. recta* [102] and to the re-classification of the former *H. caryophila* into a novel genus as ‘*Preeria caryophila*’, being more distant from *Holospora* than the other HLB genera (Fig. 1) [97]. On the other hand, ‘*Candidatus* Hafkinia simulans’ is the closest relative of *Holospora* spp. but is hosted by the brackish water peniculid ciliate *Frontonia salmastra* rather than by its close relative *Paramecium* [98]. ‘*Candidatus* Goertzia’ includes three species, namely ‘*Candidatus* Goertzia infectiva’, ‘*Candidatus* Goertzia shahrazadae’, and ‘*Candidatus* Goertzia yakutica’, respectively hosted by *P. jenningsi*, *P. multimicronucleatum*, and *P. putrinum* (Table 1) [69, 103, 104]. Further, *Holospora*/HLB are possibly awaiting to be fully characterised in association with other ciliates (e.g. *Trithigmastoma cucullulus* and *Prorodon teres*) [4]. Most of *Holospora* and HLB species were found infecting a single host species [4], but this species-specificity is not always so sharp (e.g. ‘*Preeria*’ found in species of *P. aurelia* complex as well as in *P. caudatum*) (Table 1) [97], consistent with the incongruence between bacterial and host phylogenies [96, 98]. Uncharacterised *Holospora*-related bacteria were also detected in the gut of the freshwater fish *Panaque nigrolineatus* [105] and in biofilm [106] while ‘*Candidatus* Goertzia’-like ones in the microbiome of cladocerans [35].

**Table 1:** List of described and molecularly validated *Holosporineae* species

Species	Family	Host(s)	Location(s)	Genome available	Reference
*Holospora undulata*	*Holosporaceae*	*Paramecium caudatum*	Intranuclear and infectious	Yes	[60]
*Holospora obtusa*	*Holosporaceae*	*Paramecium caudatum*	Intranuclear and infectious	Yes	[60]
‘*Holospora curviuscula*’	*Holosporaceae*	*Paramecium bursaria*	Intranuclear and infectious	Yes	[307]
‘*Holospora acuminata*’	*Holosporaceae*	*Paramecium bursaria*	Intranuclear and infectious	No	[308]
‘*Candidatus* Holospora parva’	*Holosporaceae*	*Paramecium chlorelligerum*	Intranuclear and infectious	No	[100]
‘*Candidatus* Goertzia infectiva’	*Holosporaceae*	*Paramecium jenningsi*	Intranuclear and infectious	No	[69]
‘*Candidatus* Goertzia shahrazadae’	*Holosporaceae*	*Paramecium multimicronucleatum*	Intranuclear and infectious, occasionally cytoplasmic	No	[103]
‘*Candidatus* Goertzia yakutica’	*Holosporaceae*	*Paramecium putrinum*	Intranuclear and infectious	No	[104]
‘*Candidatus* Hafkinia simulans’	*Holosporaceae*	*Frontonia salmastra*	Intranuclear and infectious	No	[98]
‘*Preeria caryophila*’	*Holosporaceae*	*Paramecium biaurelia*, *Paramecium octaurelia*, *Paramecium novaurelia*, *Paramecium caudatum*, *Paramecium* sp.	Intranuclear and infectious	No	[97]
‘*Candidatus* Fujishimia apicalis’	*Holosporaceae*	*Euplotes octocarinatus*	Cytoplasm, mostly apical	No	[52]
‘*Candidatus* Hydrogenosomobacter endosymbioticus’	*Holosporaceae*	*Cyclidium*-like scuticociliate	Cytoplasm, close to hydrogenomes	Yes	[53]
‘*Candidatus* Paraholospora nucleivisitans’	*Holosporaceae*	*Paramecium sexaurelia*	Cytoplasm and nucleus	No	[71]
‘*Candidatus* Mystax nordicus’	*Holosporaceae*	*Paramecium nephridiatum*	Cytoplasm, sometimes aggregating with mitochondria	No	[56]
‘*Candidatus* Gromoviella agglomerans’	*Holosporaceae*	*Paramecium polycaryum*	Cytoplasm, sometimes forming aggregates	Yes	[58]
‘*Candidatus* Cytomitobacter primus’	*Holosporaceae*	*Diplonema japonicum*	Cytoplasm, occasionally possibly inside mitochondria	Yes	[50]
‘*Candidatus* Cytomitobacter indipagum’	*Holosporaceae*	*Diplonema aggregans*	Cytoplasm, occasionally possibly inside mitochondria	Yes	[50]
‘*Candidatus* Cytomitobacter rhynchopi’	*Holosporaceae*	*Rhynchopus asiaticus*	Cytoplasm	No	[108]
‘*Candidatus* Ignotibacter abundans’	*Holosporaceae*	*Diplonema aggregans*	Cytoplasm	Yes	[54]
‘*Candidatus* Hepatobacter penaei’	*Holosporaceae*	*Litopenaeus vannamei* and other crustaceans	Cytoplasm	Yes	[47]
‘*Candidatus* Hepatobacter paralithodis’	*Holosporaceae*	*Paralithodes platypus*	Cytoplasm	No	[48]
‘*Candidatus* Bealeia paramacronuclearis’	*Holosporaceae*	*Paramecium biaurelia*	Cytoplasm, in proximity of the macronucleus	Yes	[1]
‘*Caedimonas varicaedens*’	‘*Caedimonadaceae*’	*Paramecium biaurelia*, *Paramecium novaurelia*, *Paramecium caudatum*, *Paramecium duboscqui*, *Spirostomum ambiguum*, *Euplotes* sp.,* Peridinium cinctum*	Cytoplasm or nucleus, depending on the host species	Yes	[7]
‘*Candidatus* Paracaedimonas acanthamoebae’	‘*Caedimonadaceae*’	*Acanthamoeba* sp.	Cytoplasm	Yes	[7]
‘*Candidatus* Nucleicultrix amoebiphila’	‘*Caedimonadaceae*’	*Hartmanella* sp.	Intranuclear and infectious	Yes	[74]
‘*Candidatus* Paracaedibacter symbiosus’	‘*Candidatus* Paracaedibacteraceae’	*Acanthamoeba* sp.	Cytoplasm	Yes	[11]
‘*Candidatus* Odyssella thessalonicensis’	‘*Candidatus* Paracaedibacteraceae’	*Acanthamoeba* sp.	Cytoplasm	Yes	[62]
‘*Candidatus* Odyssella acanthamoebae’	‘*Candidatus* Paracaedibacteraceae’	*Acanthamoeba* sp.	Cytoplasm	Yes	[11]
‘*Candidatus* Finniella lucida’	‘*Candidatus* Paracaedibacteraceae’	*Orciraptor agilis*	Cytoplasm	No	[75]
‘*Candidatus* Finniella inopinata’	‘*Candidatus* Paracaedibacteraceae’	*Viridiraptor invadens*	Cytoplasm	Yes	[75]
‘*Candidatus* Finniella dimorpha’	‘*Candidatus* Paracaedibacteraceae’	*Euplotes daidaleos*, *Euplotes eurystomus*, *Euplotes octocarinatus*	Cytoplasm	No	[52]
‘*Candidatus* Intestinibacterium nucleariae’	‘*Candidatus* Paracaedibacteraceae’	*Nuclearia delicatula*	Cytoplasm	No	[148]
‘*Candidatus* Intestinibacterium parameciiphilum’	‘*Candidatus* Paracaedibacteraceae’	*Paramecium biaurelia*	Cytoplasm	No	[95]
‘*Candidatus* Captivus acidiprotistae’	‘*Candidatus* Paracaedibacteraceae’	Unnamed protists from acidic mine drainage	Cytoplasm	No	[63]
‘*Candidatus* Parafinniella ignota’	‘*Candidatus* Paracaedibacteraceae’	*Euplotes* sp.	Cytoplasm	No	[52]
‘*Candidatus* Bodonicaedibacter vickermanii’	‘*Candidatus* Paracaedibacteraceae’	*Bodo saltans*	Cytoplasm, in proximity of the nucleus	Yes	[57]

*Holospora* and HLB display long branches in molecular phylogenies, indicative of fast sequence evolution, and their close relatives display quite long branches as well. Accordingly, the members of whole clade, including *Holospora* and HLB, have been defined as ‘fast-evolving’ *Holosporaceae* (highlighted in Fig. 1) [58], which include the majority of the characterised *Holosporaceae*. Besides holosporas, several fast-evolving *Holosporaceae* live in association with ciliates as well (Table 1) [96, 107], namely ‘*Candidatus* Fujishimia apicalis’, symbiont of *Euplotes octocarinatus* [52], ‘*Candidatus* Hydrogenosomobacter endosymbioticus’, symbiont of an anaerobic *Cyclidium*-like scuticociliate [53], ‘*Candidatus* Paraholospora nucleivisitans’, symbiont of *Paramecium sexaurelia* [71], ‘*Candidatus* Mystax nordicus’, symbiont of *Paramecium nephridiatum* [56], and ‘*Candidatus* Gromoviella agglomerans’, symbiont of *Paramecium polycaryum* [58]. Other members of this clade were characterised in association with marine diplonemids (Table 1), namely ‘*Candidatus* Cytomitobacter primus’, symbiont of *Diplonema japonicum* [50], ‘*Candidatus* Cytomitobacter rhynchopi’, symbiont of *Rhynchopus asiaticus* [108], as well as ‘*Candidatus* Cytomitobacter indipagum’ and ‘*Candidatus* Ignotibacter abundans’ (formerly ‘*Candidatus* Nesciobacter abundans’), symbionts of the same strain of *Diplonema aggregans* [54]. Uncharacterised fast-evolving *Holosporaceae* were found in association with the prairie dog flea *Oropsylla hirsuta* [29], in the gut of the hemipteran *Pyrrhocoris apterus* [89], and in the microbiome of the marine bryozoon *Bugula neritina* [33]. Other members of this clade were detected in multiple environments and sources, such as freshwater lakes [17, 22, 24, 109], hypersaline microbial mat [18], activated sludge [110], acidic mine drainage [25], ocean depths [111], soil [112], and hospital dental units [113].

The genus ‘*Candidatus* Hepatobacter’ is phylogenetically proximate to the fast-evolving *Holosporaceae* (Fig. 1). ‘*Candidatus* Hepatobacter penaei’ thrives inside the epithelial cells of the hepatopancreas of the marine shrimp *Litopenaeus vannamei* (Table 1) [10, 47, 49] and possibly other akin crustaceans, such as *L. setiferus*, *L. stylirostris*, *Farfantepenaeus aztecus*, and *F. californiensis* [114]. On the other hand, ‘*Candidatus* Hepatobacter paralithodis’ was found in the hepatopancreatic epithelium of the crab *Paralithodes platypus* [48]. Phylogenetically close bacteria were found in possible association with amphipods (*Melita plumosa*) [115], sponges (*Xestospongia muta*) [31], and oribatid mites (*Achipteria coleoptrata*) [34]. Thus, notwithstanding the uncertainties on the actual hosts for the latter bacteria, it is possible to wonder whether the members of this subclade could present a marked preference towards arthropod and other metazoan hosts rather than protists (Fig. 1), being an exception among the whole *Holosporineae*.

Early diverging *Holosporaceae* are characterised by overall shorter branches in molecular phylogenies. The only described species is ‘*Candidatus* Bealeia paramacronuclearis’, found as symbiont of two *P. biaurelia* strains (Fig. 1; Table 1), in one case in coexistence with the *Rickettsiales* bacterium ‘*Candidatus* Fokinia cryptica’ [1]. ‘*Candidatus* Bealeia’-allied bacteria were found in freshwater particles associated with blooms of dinoflagellate *Alexandrium monilatum* [116]. Other early-diverging *Holosporaceae* were detected from various origins, including the stomach of the catfish *Pelteobagrus fulvidraco* [90], the intestine of zebrafish [88], aquatic moss [117], soil [118–122], mine drainage and water [16, 123], freshwater and marine sediments [124–126], and volcanic cinder deposit [127].

Further sequences assigned to *Holosporaceae*-related bacteria were found in the microbiota of *Daphnia* cf. *pulex* [37] and in olive oil production pomace [128].

The ‘*Caedimonadaceae*’ are hosted by diverse protists, such as ciliates, dinoflagellates, and amoebas [7, 11, 74, 129]. This is the least rich family in terms of described genera and species, including only three monotypic genera (Fig. 1; Table 1). ‘*Caedimonas varicaedens*’ is the most-studied member. This bacterium was re-described recently [7], but it was known for decades, being previously placed within the gammaproteobacterial genus *Caedibacter*, due to shared traits such as R-bodies and killer trait [7]. Former species now part of ‘*Caedimonas varicaedens*’ were *Caedibacter varicaedens*, *Caedibacter caryophilus*, and ‘*Caedibacter macronucleorum*’ [67, 130, 131]. ‘*Caedimonas*’ was found as symbiont of multiple *Paramecium* species, in particular, *P. caudatum*, members of the *P. aurelia* complex such as *P. biaurelia* and *P. novaurelia*, and *P. duboscqui*, being the causative agent of the killer effect [6, 67, 131–133]. On the other hand, it was also detected as symbiont of other ciliates (Table 1), such as *Spirostomum ambiguum* [134] and *Euplotes* sp. [52], as well as in the dinoflagellate *Peridinium cinctum* [6, 129], though without evidence of a killer effect. Further relatives of ‘*Caedimonas*’ were retrieved from various origins, such as urban aerosol [135] and sludge [136]. ‘*Candidatus* Paracaedimonas acanthamobae’ (formerly ‘*Candidatus* Caedibacter acanthamoebae’ [11]) is phylogenetically close to ‘*Caedimonas*’ and was originally described as symbiont of *Acanthamoeba* [11]. ‘*Candidatus* Paracaedimonas’ bacteria were repeatedly detected in acanthamoebas worldwide [137, 138] and were also found in soil [139] and bioreactors [140]. Moreover, sequences of bacteria more distantly allied to ‘*Caedimonas*’ and ‘*Candidatus* Paracaedimonas’ were retrieved from drinking water [15].

The other and more diverging described representative of the ‘*Caedimonadaceae*’ is ‘*Candidatus* Nucleicultrix amoebiphila’ (Fig. 1; Table 1), symbiont of the amoebozoan *Hartmanella* sp. [74]. This bacterium displays an infectious cycle, as was able to invade *Acanthamoeba castellanii* in laboratory experiments. Sequences of bacteria that are phylogenetically allied to ‘*Candidatus* Nucleicultrix’ were found in soil [141, 142] and in a lake [143].

Further members of the ‘*Caedimonadaceae*’ were detected in the gut of the beetle *Harpalus pensylvanicus* [86], as well as in lakes [26], seawater [144], marine sediments [145, 146], and drinking water [147].

The family ‘*Candidatus* Paracaedibacteraceae’ displays a high phylogenetic diversity, with seven genera and 11 species described (Fig. 1; Table 1). The breadth of their collective host range is comparable to the *Holosporaceae* and possibly even wider in terms of relative frequency of each main host lineage. Ascertained hosts are protists belonging to various lineages, including amoebas, cercozoans, ciliates, nucleariids, and euglenozoans [11, 57, 62, 69, 75, 95, 148]. The most long-term known representatives of the family belong to the genus ‘*Candidatus* Paracaedibacter’ and are found intracellularly in *Acanthamoeba*, including clinical isolates [11, 149–152], which led some authors wondering about their role in pathogenicity for the eye of the amoebas. ‘*Candidatus* Paracaedibacter’ is paraphyletic with respect to ‘*Candidatus* Odyssella thessalonicensis’, which is a symbiont of *Acanthamoeba* as well [62] and is more closely related to ‘*Candidatus* Paracaedibacter acanthamoebae’ than the two of them to ‘*Candidatus* Paracaedibacter symbiosus’ (Fig. 1) [1, 75]. Specifically, ‘*Candidatus* Paracaedibacter acanthamoebae’ and ‘*Candidatus* Odyssella thessalonicensis’ share a 16S rRNA gene identity of 97.8%, while, respectively, having 92.5% and 91.8% identity with ‘*Candidatus* Paracaedibacter symbiosus’ [1]. Considering the commonly accepted genus threshold for the 16S rRNA gene (94.5%) [153], and that the original description did not designate any type species for the genus ‘*Candidatus* Paracaedibacter’ [11], we propose to elect ‘*Candidatus* Paracaedibacter symbiosus’ as type and to move ‘*Candidatus* Paracaedibacter acanthamoebae’ into the genus ‘*Candidatus* Odyssella’ as ‘*Candidatus* Odyssella acanthamoebae’ comb. nov. (Table 1) and we will be referr to this bacterium as such from now on (see taxonomic revision at the end). Relatives of ‘*Candidatus* Paracaedibacter’/ ‘*Candidatus* Odyssella’ were detected in many environments and sources, namely lakes [22, 24], soil [154, 155], groundwater [156, 157], drinking water [23, 158], sludge [159, 160], wastewater [161], acidic mine drainage [25], textiles [162], human faeces [163], hospital dental units [113], and hydrocarbon [164].

The ‘*Candidatus* Paracaedibacteraceae’ includes several other clades with respective phylogenetic relationships not yet fully and consistently resolved (Fig. 1). ‘*Candidatus* Finniella’ bacteria belong to one of those clades and are hosted by cercozoans, namely ‘*Candidatus* Finniella lucida’ symbiont of *Orciraptor agilis* and ‘*Candidatus* Finniella inopinata’ of *Viridiraptor invadens* [75], and by ciliates, namely ‘*Candidatus* Finniella dimorpha’ symbiont of multiple *Euplotes* spp. (Table 1) [52]. Relatives of ‘*Candidatus* Finniella’ were retrieved in a possible association with *Hydra vulgaris* [70] and from multiple additional sources, such as lakes/rivers [21, 24, 27, 165], drinking water [23, 166], acidic mine drainage [16, 25], and electronic waste aerosol [167].

Another clade of the ‘*Candidatus* Paracaedibacteraceae’ is the one encompassing the genus ‘*Candidatus* Intestinibacterium’ (formerly ‘*Candidatus* Intestinusbacter’) (Fig. 1; Table 1), with the described species ‘*Candidatus* Intestinibacterium nucleariae’ symbiont of the opisthokont *Nuclearia delicatula* [148] and ‘*Candidatus* Intestinibacterium parameciiphilum’ symbiont of *P. biaurelia* [95]. Members of the same clade were retrieved from several, prevalently aquatic, sources, such as lakes [17, 19–21, 24, 26, 27, 168, 169], a river [170], seawater [143], a sulphidic spring [171], subsurface water [172], drinking water [173], wastewater [161], biofilm [157, 174], microbial mat [175], and even human skin [91, 94]. Moreover, bacteria related to both ‘*Candidatus* Intestinibacterium’ and ‘*Candidatus* Finniella’ were retrieved in an aquaculture after applying silver nanoparticles [176].

‘*Candidatus* Captivus acidiprotistae’ is another representative of the family (Table 1), found as endosymbiont in unnamed protists from an acidic mine drainage [63]. A close relative is an unnamed symbiont of the euglenozoan *Petalomonas sphagnophila* (Fig. 1) [72]. Further relatives were found in soil [177–179], sediment [180], acidic mine drainage [181], and an acidic pit lake [169]. The other two described members of the ‘*Candidatus* Paracaedibacteraceae’ are ‘*Candidatus* Parafinniella ignota’, symbiont of *Euplotes* ciliates [52] in coexistence with other intracellular bacteria [182], and ‘*Candidatus* Bodonicaedibacter vickermanii’ (formerly ‘*Candidatus* Bodocaedibacter vickermanii’), symbiont of the free-living kinetoplastid *Bodo saltans* (Fig. 1; Table 1) [57].

Another quite conspicuous clade of ‘*Candidatus* Paracaedibacteraceae’ includes only uncharacterised bacteria, frequently derived from the gut of various animals (Fig. 1), in particular termites (e.g. *Reticulitermes speratus*, *R. santonensis*, and *Coptotermers curvignatus*) [28, 32, 183–188], as well as the sea cucumber *Apostichopus japonicus* [36], the fish *Ctenochaetus striatus* [189], and springbok antelopes [87].

Further ‘*Candidatus* Paracaedibacteraceae’ bacteria were retrieved from human skin [93], the blowhole of the bottlenose dolphin *Tursiops truncatus* [92], the epithelium of *Hydra magnipapillata* [30], lakes [21, 24, 190], a river [191], biofilm [192, 193], drinking water [147, 194], groundwater [195], and wastewater [196, 197].

## Host Interactions, Genomics, and Evolution

The interactions between the *Holosporineae* and their hosts, both in terms of mechanisms and effects, are overall still poorly understood, also due to the inherent experimental limitations in handling host-associated bacteria. In the last 15 years, a growing number of *Holosporineae* genomes have been sequenced, belonging to each of the three families, namely *Holosporaceae*, three ‘*Caedimonadaceae*’, and five ‘*Candidatus* Paracaedibacteraceae’ (Table 2). While many genus-level sublineages are not yet sequenced, available genomes have represented a major advance, allowing a deeper understanding of the functional features of the *Holosporineae* and of their evolution. Leveraging on the available observational, experimental, and genomic data, below, we will present a comprehensive account of the current knowledge on those subjects, focusing in particular on the most-studied cases, namely *Holospora*, ‘*Caedimonas*’, and the NHP determinant ‘*Candidatus* Hepatobacter penaei’.

**Table 2:** List of *Holosporineae* with assembled genomes, and respective assembly statistics

Species	Host	Size	GC%	Number of contigs/scaffolds	N50	Plasmid number	Accession	Reference
Family *Holosporaceae*								
*Holospora undulata* subsp. *undulata*	*Paramecium caudatum*	1.4 Mbp	36	203	10.9 kbp	Not determined	GCA_000388175.3	[226, 309]
*Holospora undulata* subsp. *elegans*	*Paramecium caudatum*	1.3 Mbp	36	152	13 kbp	Not determined	GCA_000648275.1	[226]
*Holospora obtusa*	*Paramecium caudatum*	1.3 Mbp	35	91	24.4 kbp	Not determined	GCA_000469665.2	[226]
‘*Holospora curviuscula*’	*Paramecium bursaria*	1.7 Mbp	37.5	152	40.5 kbp	Not determined	GCA_002930195.1	[101]
‘*Candidatus* Hydrogenosomobacter endosymbioticus’	*Cyclidium*-like scuticociliate	826.7 kbp	41.5	1	826.7 kbp	Absent	GCA_021654655.1	[310]
‘*Candidatus* Cytomitobacter primus’	*Diplonema japonicum*	622.4 kbp	30	1	622.4 kbp	Absent	GCA_008189405.1	[54]
‘*Candidatus* Cytomitobacter indipagum’	*Diplonema aggregans*	628 kbp	29.5	1	628 kbp	Absent	GCA_008189285.1	[54]
‘*Candidatus* Ignotibacter abundans’	*Diplonema aggregans*	616.1 kbp	30	1	616.1 kbp	Absent	GCA_008189525.1	[54]
‘*Candidatus* Gromoviella agglomerans’	*Paramecium polycaryum*	590 kbp	32	1	590 kbp	Absent	GCA_021065005.1	[58]
‘*Candidatus* Hepatobacter penaei’	*Litopenaeus vannamei*	1.1 Mbp	50	5	334.2 kbp	Not determined	GCA_000742475.1	[78]
‘*Candidatus* Bealeia paramacronuclearis’	*Paramecium biaurelia*	1.9 Mbp	43	1	1.9 Mbp	6	GCA_036670005.1	[14]
Family ‘*Caedimonadaceae*’								
‘*Caedimonas varicaedens*’	*Paramecium biaurelia*	1.7 Mbp	42	142	20.2 kbp	Not determined	GCA_001192655.1	[311]
‘*Candidatus* Paracaedimonas acanthamoebae’	*Acanthamoeba* sp.	1.7 Mbp	38	1	1.7 Mbp	5	GCA_000743035.1	[78]
‘*Candidatus* Nucleicultrix amoebiphila’	*Hartmanella* sp.	1.8 Mbp	39.5	1	1.8 Mbp	Absent	GCA_002117145.1	Schulz et al., unpublished
Family ‘*Candidatus* Paracaedibacteraceae’								
‘*Candidatus* Paracaedibacter symbiosus’	*Acanthamoeba* sp.	2.7 Mbp	41	50	1.7 Mbp	2	GCA_000757605.1	[78]
‘*Candidatus* Odyssella thessalonicensis’	*Acanthamoeba* sp.	2.8 Mbp	42	20	388.1 kbp	Not determined	GCA_000190415.2	[76]
‘*Candidatus* Odyssella acanthamoebae’	*Acanthamoeba* sp.	2.5 Mbp	41	1	2.5 Mbp	1	GCA_000742835.1	[78]
‘*Candidatus* Finniella inopinata’	*Viridiraptor invadens*	1.8 Mbp	44	28	174.7 kbp	Not determined	GCA_004210305.1	[59]
‘*Candidatus* Bodonicaedibacter vickermanii’	*Bodo saltans*	1.4 Mbp	40.5	1	1.4 Mbp	Absent	GCA_014896945.1	[57]

**Fig. 2:**
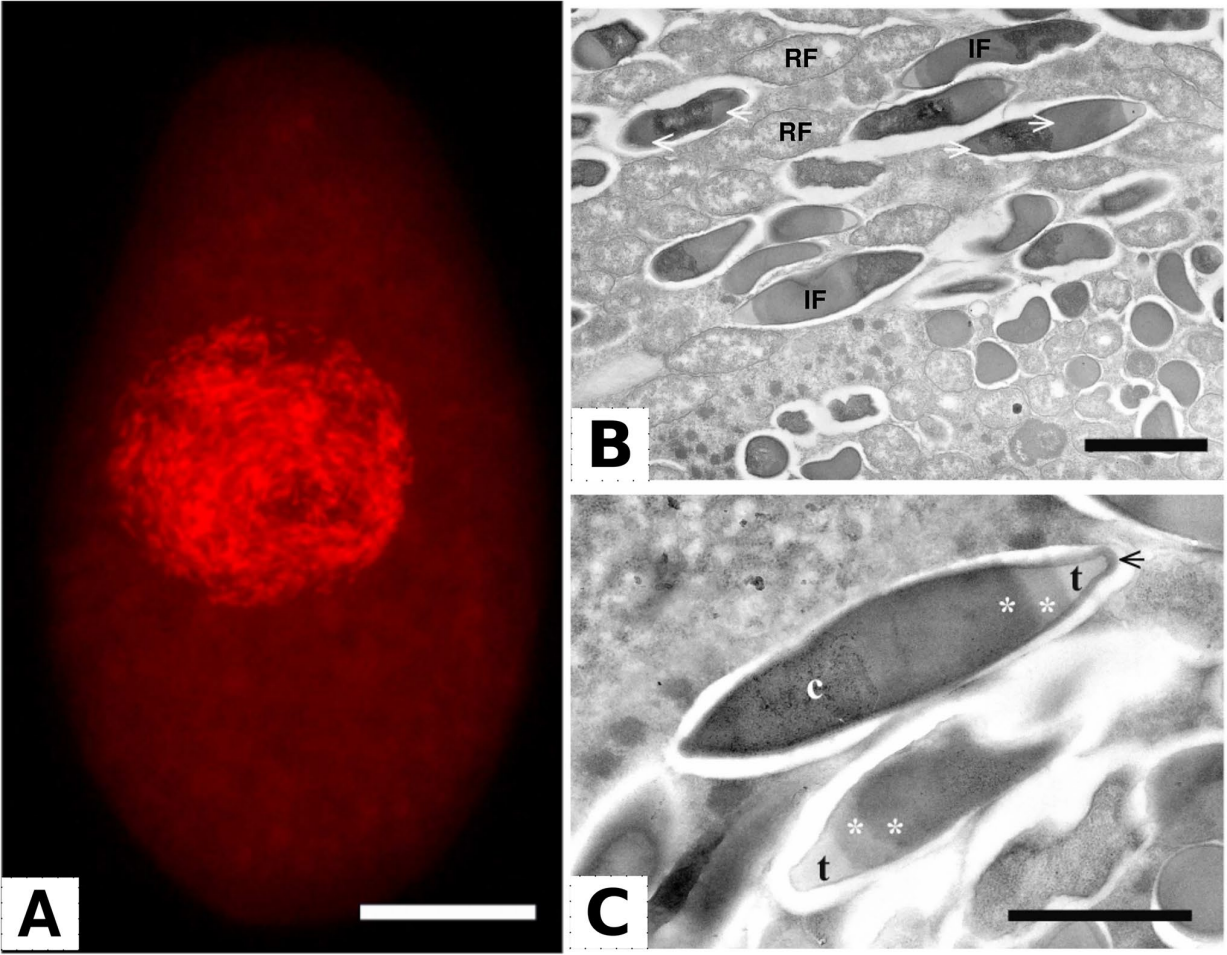
Subcellular location and ultrastructure of ‘*Candidatus* Holospora parva’, symbiont of the ciliate *P. chlorelligerum*, modified from [100]. **A** Densely packed bacteria inside the macronucleus of the host, stained with a red fluorescent probe specific for ‘*Candidatus* Holospora parva’. **B, C** Ultrastructure of the bacteria. In **B**, both reproductive forms (RF) and infectious forms (IF) are shown, with the white arrows indicating the enlarged periplasm of the latter form. In **C**, a magnification of the details of an infection form is presented, namely the cytoplasm (c), the periplasm (asterisks), and its apical tip (t). The black arrow indicates fine fibrous material that may be present on the surface of some infectious forms of this bacterium. Scale bars: **A** 20 μm; **B** 2 μm; **C** 1.5 μm

*Holospora* and HLB are probably the most deeply investigated among the *Holosporineae* and have been subject of dedicated extensive reviews over the years [2–4]. Indeed, these bacteria present a distinct set of apomorphic features, which allow them to engage in a dimorphic infectious life cycle interacting with their ciliate hosts, which in most of the characterised cases, though not all [98], belong to genus *Paramecium* [4, 69, 97, 100, 104]. The reproductive form of the typical holosporas is shaped as a short Gram-negative rod [69, 198, 199] and actively multiplies within the host nucleus, which may be the micronucleus or, more frequently, macronucleus of the ciliate, depending on the bacterial and host species (Fig. 2) [2, 4]. Under starvation or other conditions, which may be linked to the arrest of host protein synthesis [2, 200], the reproductive form differentiates into an elongated infectious form, which presents an extended electron-dense periplasm with a translucent tip (Fig. 2) [4, 69, 198, 200, 201]. The infectious forms do not divide and are typically released into the external medium through exocytic vesicles [202–206], formed by different mechanisms according to the species [207]. In extreme cases, infectious forms may cause the host cell lysis, possibly as a consequence of the deliverance of lipopolysaccharide, thus leading to their own release [2]. The infectious form is able to survive apart from the host for several days [208]. If ingested by a novel host cell, it gets activated by the acidification of the host digestive vacuole and is able to escape from the vacuole with the periplasmic tip ahead and then interacting with the host membrane trafficking systems and cytoskeleton, in order to reach its target nucleus [209–214]. It can enter the nucleus without causing its disruption and therein will produce back novel reproductive forms, thus fuelling the *Holospora* life cycle [2, 3, 215, 216].

The molecular determinants of the infection are only partly understood. Some studies, mainly on *H. obtusa*, were aimed at the characterisation of stage-specific proteins and their possible involvement in different infection phases, such as binding to actin or to the macronucleus [212, 217–224]. The identified proteins have little homology with those of other organisms and were labelled by their molecular weight (e.g. 89 kDa, 63 kDa, or 5.4 kDa proteins), thus preventing more extensive comparative studies to date.

As described above, *Holospora* and HLB present the typical traits of specialised parasites, namely an infectious life cycle with the possibility to harm their hosts, including hampering sexual processes [206, 225]. An ongoing parasitic interaction is also supported by genomic evidences of quite pronounced scavenging of metabolites by the bacteria from their hosts [101, 226], as well as by the presence of host resistance mechanisms of the ciliates against the infection [227–231]. As such, *Holospora*/HLB and *Paramecium* have been repeatedly used as experimental models for the evolution of host-parasite interactions, including plasticity and trade-offs between transmission modes, infectivity, and virulence in the parasite [232–235], as well as between resistance and fitness in the host [236]. Further studies investigated local adaptation [237–239], effect of environmental variations [240–243], impact of host growth and lifespan on the growth, infectivity and virulence of the parasite [244, 245], reciprocal effects of parasite traits and host dispersal [246–249], and competition among parasites [250]. At the same time, the effect of *Holospora* and the interplay with its host are more complex than a purely parasitic interaction. Indeed, in particular as a reproductive form, it was shown to induce positive effects on the host, namely protection against environmental stress, such as temperature, salinity, and osmotic variations [2, 251–253]. This has been tentatively linked to an enhanced heat-shock protein expression both of the host and the bacterium, which could make the host more reactive to stressors [254–256]. These findings could explain the observation of a higher frequency of bacteria in hosts sampled from brackish with respect to freshwater environments [134].

In any case, the remarkable traits of the interaction between *Holospora*/HLB and *Paramecium/*other ciliates are suggestive of a significant (co)evolutionary specialisation, which however can be difficult to trace by comparative analyses, considering the sharp differences with respect to the other closely-related *Holosporineae*. Nevertheless, it seems interesting to notice two different cases, which show somehow intermediate traits and could thus be seen as potentially reminiscent of some ancestral steps in the evolution of the peculiar infectious nuclear tropism. The first case is the HLB ‘*Candidatus* Goertzia sharazharadae’, which, besides being resident within the host macronucleus as typical for this bacterial lineage, was recurringly found free (i.e. without being enclosed in host-derived membranes) in the host cytoplasm [103]. The other instance is the one of ‘*Candidatus* Paraholospora nucleivisitans’, a fast-evolving *Holosporaceae* bacterium that is closely related to *Holospora* and HLB clade, although not its direct sister lineage. ‘*Candidatus* Paraholospora’ was observed in the host cytoplasm or, alternatively, in the nucleus, otherwise more rarely in both locations in the same host cell [71].

It is worth considering that many other *Holosporaceae* and *Holosporineae* in general display some relation with host organelles including the nucleus, up to being intranuclear as well. The early-diverging *Holosporaceae* bacterium ‘*Candidatus* Bealeia paramacronuclearis’ is loosely co-localised with the host macronucleus from its outside [1], similarly to the position of the ‘*Candidatus* Paracaedibacteraceae’ bacterium ‘*Candidatus* Bodonicaedibacter vickermanii’ with respect to its host nucleus [57]. On the other hand, the fast-evolving *Holosporaceae* bacteria ‘*Candidatus* Cytomitobacter primus’ and ‘*Candidatus* Mystax nordicus’ were found in proximity or even aggregation with host mitochondria [50, 56], with the former bacterium possibly able to enter within these organelles. Moreover, both ‘*Candidatus* Mystax’ and ‘*Candidatus* Gromoviella agglomerans’ (also a member of fast-evolving *Holosporaceae*) can form bacterial aggregates within the respective host cells [56, 58], the latter also with potential lethal division effects on the host. Finally, ‘*Candidatus* Hydrogenosomobacter endosymbioticus’ was found in association with host hydrogenosomes [53].

The endonuclear localisation is not exclusive of the *Holosporaceae* among the *Holosporineae*, as the same condition is typical also of some members of the ‘*Caedimonaceae*’. For instance, the intracellular localisation of ‘*Caedimonas*’ appears to be correlated with the host species, being intramacronuclear in *Paramecium caudatum* and *Paramecium duboscqui* [130, 131], while cytoplasmic in the species of the *P. aurelia* complex [7, 132]. The case of ‘*Candidatus* Nucleicultrix amoebiphila’ is even more distinctive, since this bacterium presents a complex infectious life cycle that is highly reminiscent of the one of *Holospora* and HLB [74]. Similarly to the latter, the effect of ‘*Candidatus* Nucleicultrix’ on the host is variable and was shown to be negligible for its natural host, the amoeba *Hartmanella*, but lethal for experimentally infected *Acanthamoeba castellanii*. Overall, it seems legitimate to speculate that the ability for interacting with host nuclei could have been ancestral in the *Holosporaceae* + ‘*Caedimonadaceae*’ lineage, being successively lost (or remaining unnoticed to date) in some of the descendants. Alternatively, multiple parallel evolutionary events of this trait are also possible, considering its independent evolution in other phylogenetically unrelated bacteria [257]. On the other hand, and remarkably, it seems more parsimonious to infer that the highly specialised infectious life cycles of *Holospora* + HLB and of ‘*Candidatus* Nucleicultrix’ have most likely arisen independently from such a hypothetical ‘permissive’ ancestral ability to colonise host nuclei.

**Fig. 3:**
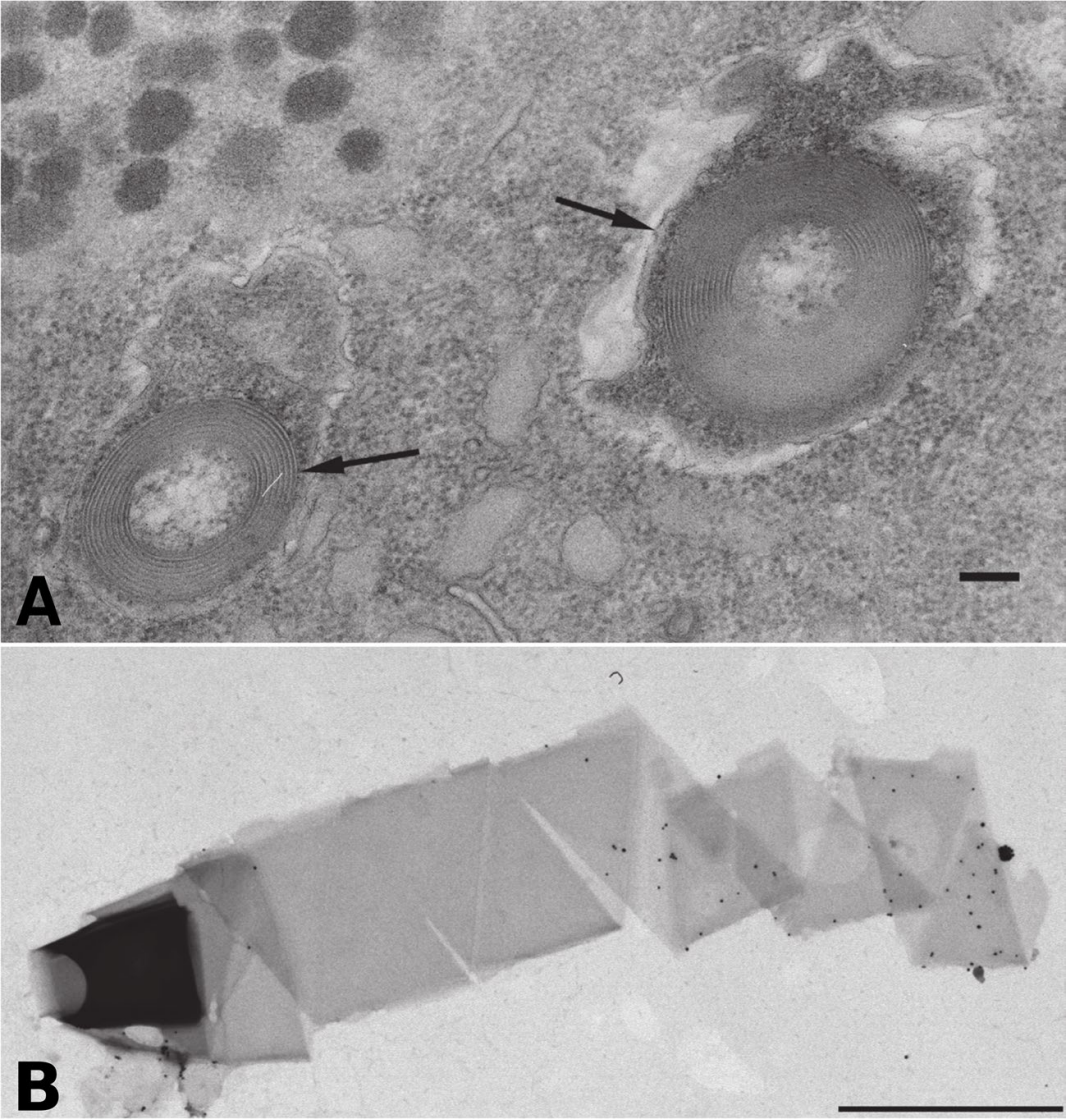
Ultrastructure of R-bodies, modified from [306]. **A** Coiled R-bodies (black arrows) within the bacterial symbiont cells in the *Paramecium* cytoplasm. **B** Isolated R-body in the process of unrolling in a telescopic fashion. Scale bars: **A** 100 nm; **B** 1 μm

‘*Caedimonas varicaedens*’, the other deeply investigated member of the *Holosporineae*, has been studied particularly for the distinctive killer trait that it confers to its *Paramecium* hosts, similarly to the gammaproteobacterium *Caedibacter taeniospiralis* [5–7]. A decades-long history of investigations was focused on the killer trait and its determinants, also before the discovery that ‘*Caedimonas*’ and *Caedibacter* were phylogenetically apart*.* As such, these two bacteria were treated jointly in many studies and in reviews on the subject [6, 258, 259], which will be summarised below. Part of the intracellular bacterial population ceases to divide and produce proteinaceous coiled ribbons (Fig. 3) that are light-refractile and thus called R-bodies [260, 261]. The bacteria bearing R-bodies can be discharged extracellularly through their host cytoproct [262] and, when ingested by a novel host, are lysed in the digestive vacuoles leading to the release of the R-bodies, which under those acidic conditions unroll and break the vacuolar membrane (Fig. 3) [261, 262]. The unrolling of R-bodies ultimately leads to host death, with different lethal symptoms according to the bacterial genotype, including reversion of the normal rotation direction of the cell, formation of large vacuoles, or paralysis [258]. Although required for the killer trait [263], the R-bodies are not directly toxic, rather their role is in the disruption of the host vacuolar membrane, allowing the delivery of the actual toxin produced by the bacterium [264–266]. This toxin is probably a protein but was yet not conclusively identified [6, 259]. The potential toxic activity of ‘*Caedimonas*’ towards eukaryotes other than *Paramecium* has been only seldom explored until now, with still inconclusive evidence [267]. On the other hand, paramecia hosting ‘*Caedimonas*’ are resistant, probably thanks to an antidote produced by the bacterium. Interestingly, in both ‘*Caedimonas*’ and *Caedibacter*, the determinants of R-bodies (as well as probably toxin and antidote) are encoded on plasmids and/or linked to phage particles and prophage induction [260, 268–272]. As such, the multipartite interactions involving ciliates, bacteria, and plasmids/phages were meaningfully defined as involving ‘extrachromosomal elements of extrachromosomal elements of *Paramecium*’ [273]. Being encoded on mobile elements, R-bodies and other determinants of the killer trait are likely horizontally transmissible, which would explain their presence in unrelated bacteria, such as *Caedibacter* and ‘*Caedimonas*’, as well as others (see below).

Thanks to the killer trait, the paramecia bearing ‘*Caedimonas*’ display competitive advantages [133, 274], but at the same time, the bacteria can be parasitic for taking ATP for energy and other metabolites from their hosts [130, 274, 275], somehow comparably to *Holospora*. Besides, the peculiar effect of the killer trait was tentatively envisioned as a phenomenon of addictive manipulation, namely a way by which the bacterium indirectly prevents the host from getting rid of it, by ‘punishing’ it thanks to the action of bacteria released by still-infected neighbouring host cells [43]. This is posited to be analogous to the reproductive manipulation of arthropods exerted by *Wolbachia* and other bacteria, in particular cytoplasmic incompatibility, by which infected males sterilise the crosses with uninfected females, thus favouring the reproduction of infected females, the only ones that transmit the bacteria to the progeny [276]. Following these lines of thought, it is worth to consider that, although R-bodies and their genetic determinants are best studied in *Caedibacter* and ‘*Caedimonas*’, they are found in a wide range of phylogenetically unrelated bacteria [258, 277], which can employ their R-bodies in interaction with eukaryotes [278–281]. Interestingly, these other bacteria equipped with R-body genes include two other *Holosporineae*, affiliated to different families, namely ‘*Candidatus* Bealeia paramacronuclearis’ (*Holosporaceae*) and ‘*Candidatus* Finniella inopinata’ (‘*Candidatus* Paracaedibacteraceae’) [14]. R-bodies were not observed in either of these bacteria [1, 75], suggesting that their expression is conditional, and leaving still open which is their role (if any) in the interaction with the respective hosts, in particular, a possible addictive manipulation alike to the one exerted by ‘*Caedimonas*’ through the killer trait. Herein, it seems worthwhile to consider the potential implications in the evolution of *Holosporineae* as a whole, since phylogenetic reconstructions of the R-body genes are compatible both with a vertical inheritance from the ancestor of this lineage (followed by losses in other representatives) or with a recent exchange between the equipped members [14].

The other more investigated case study among the *Holosporineae* is ‘*Candidatus* Hepatobacter penaei’ (*Holosporaceae*), known for being the causative agent of NHP [49]. NHP is mostly known in the Pacific white shrimp *L. vannamei*, which is the main farmed shrimp worldwide [282], but the disease (or its causative agent) was detected also in other shrimp species, such as *L. setiferus*, *L. stylirostris*, *F. aztecus*, *F. californiensis*, *F. duorarum*, *Penaeus monodon*, *Fenneropenaeus merguensis*, and *Melicertus marginatus*, as well as the American lobster *Homarus americanus* [49, 114]. ‘*Candidatus* Hepatobacter penaei’ resides and multiplies exclusively inside the tubular epithelial cells of the host hepatopancreas [283], and its involvement in NHP was experimentally demonstrated [284]. This bacterium is pleomorphic, being observed as a coccoid/short rod-shaped form, and as a long helical rod with eight long periplasmic flagella [285, 286], which might be involved in bacterial motility, adherence to host cells, and virulence [287]. The bacterial infection heavily damages the hepatopancreas, causing detachment of tubular cells, melanisation, and necrosis of the tubules, strong intracellular haemocytosis, and oedema [49]. The disease ultimately affects multiple organs and functions, as the observed signs include, among many others, anorexia, lethargy, abdominal muscular atrophy, soft exoskeleton, decreased growth rate, empty intestines, erosion of appendages, darkening, and lesions in the cuticle [288, 289]. NHP chronically causes mortalities of up to 50 − 95% in affected postlarval stages and well as in juveniles and broodstock [290], with significant economical impacts in terms of production losses and management costs [291]. At the appropriate progression stages, it can be effectively counteracted by antibiotic treatments [292]. The transmission of the NHP is not entirely clarified but can occur rapidly in densely populated farms [49], and a possible role of microalgae, other crustaceans (*Artemia*), or zooplankton as vectors was hypothesised [293, 294]. The disease is common especially in the south of the USA, as well as in Central and South America, and was shown to occur typically after persistently high water temperature (29–35 °C) and salinity (30–40‰) during summer, with the bacterium reaching over 15% prevalence in farms [295], but less than 1% in the wild [282]. Available molecular diagnostic tools include a multilocus sequence analysis [114], as well as a qPCR on *flgE* flagellar gene [290].

It is noteworthy that the recently described relative ‘*Candidatus* Hepatobacter paralithodis’ also affects a crustacean, namely the blue king crab *P. platypus*, although with very low prevalence in the wild [48]. Several traits are in common with NHP, as this bacterium as well is localised only within the epithelial cells of the host hepatopancreas, mostly free from host vacuoles. Two main morphotypes are present, namely rounded forms, often found in chains, and elongated rods devoid of flagella. The hepatopancreas structure and function are impaired by the bacteria, with hypertrophy and desquamation of infected cells, softening of tubules, granuloma, and necrosis. However, very little external signs of the disease were observed, besides lethargy. The geographical and climatic pattern is different from NHP, as ‘*Candidatus* Hepatobacter paralithodis’ was found in a much colder area of the Northern Hemisphere (Sea of Okhotsk), but seasonal patterns in the disease emergence were suspected as well [48].

Taking into account the more deeply investigated cases presented above and the available data on other representatives, it is thus possible to summarise the available knowledge into an evolutionary scenario for the interactions between the *Holosporineae* and their hosts. In quite ancient times, the free-living ancestors of this bacterial lineage acquired the ability to interact with eukaryotic hosts, which were most likely unicellular aquatic ones, and, based on relative environmental frequency of the current available representatives and its phylogenetic patterns, may have been more specifically freshwater [95]. It seems likely that, analogous to other professional symbionts [296–299], secretion systems and effectors have likely played a pivotal role in the establishment and successive development of these interactions. In the case of the *Holosporineae*, the most credible candidate is the type VI secretion system, conserved in almost all the genomes sequenced, including the smallest ones, with the exception of *Holospora* spp. Despite the lack of experimental data on its functioning and on possible secreted molecules, this high conservation is highly indicative of the key role of this apparatus in the lifestyle of *Holosporineae*. The type VI secretion system of *Holosporineae* is probably a non-standard one, considering the apparent lack of genes for some main components (outer membrane complex TssD and inner tube TssJ) [54, 58]. The case of *Holospora* could be explained by the markedly specialised infectious life cycle of these bacteria [2, 3], which likely involves equally specialised effector molecules [212, 220, 222, 224], possibly making the ‘canonical’ ones among the *Holosporineae* superfluous.

As a matter of fact, while only few members of the *Holosporineae* were experimentally shown to invade novel hosts [3, 74, 284], indirect evidence from compared host and symbiont phylogenies clearly indicates the recurrent ability of these bacteria of host transfer and host species shift along their evolutionary history, e.g. [1, 14, 52, 54, 56, 75]. Flagella likely play an important role in transmission and invasion of novel hosts, as hypothesised for other professional symbionts [40, 300–302], potentially working also as an additional secretion system [303]. As described above, a role of flagella in host invasion could be the case for ‘*Candidatus* Hepatobacter penaei’, which is the only *Holosporineae* bacterium observed bearing flagella but likely applies also for several other representatives equipped with flagellar genes, which could express them conditionally, similarly to what many *Rickettsiales* are thought to do [40, 300]. From an evolutionary perspective, a still relevant open point is the transition from putative ancestral protist hosts to Metazoans. This is a common trait observed among professional symbionts, having significant medical or veterinary impacts, as it can pave the way for the emergence of dangerous pathogens [44–46]. In the case of the *Holosporineae*, this has occurred relatively rarely, with the only ascertained documented case being the ‘*Candidatus* Hepatobacter’ lineage [47, 48]. It is yet to be determined whether this is only somehow accidental, or whether the molecular genetic set of the *Holosporineae* is for any reason less permissive than other professional symbiont lineages for such transition.

Comparative genomics quite sharply indicates metabolic dependence of the *Holosporineae* on their hosts, particularly in terms of obtaining metabolic precursors, such as amino acids or nucleotides, and cofactors [14]. With respect to their free-living ancestors, all the *Holosporineae* appear to have experienced variable degrees of genome reduction, from relatively large sizes (2.5–3 Mb) in certain ‘*Candidatus* Paracaedibacteraceae’ [76, 78], down to less than 600 kbp in some fast-evolving *Holosporaceae* [54, 58]. This suggests that the reduction was probably rather gradual and/or recent, with possible lineage-specific patterns, with the larger gene repertoires suggestive of more complex yet uncharacterised regulatory mechanisms and interaction mechanisms with the hosts. Reductive trends are particularly marked among the *Holosporaceae*, likely with concurrent specialisation towards certain life cycles and/or hosts, in particular in the extremely reduced fast-evolving members. Indications of possible multiple losses of a biosynthetic ability (i.e. for biotin) as a consequence of the independent acquisition of the respective transporters in different sub-lineages of *Holosporineae* were obtained [14], reminiscently of the *Rickettsiales* [40]. Nevertheless, the data available so far suggest that the major evolutionary steps which resulted in host-dependence occurred only once in the common ancestral evolution of all *Holosporineae*, rather than independently in the different sub-lineages.

Regarding interaction mechanisms and effects on the host, the presence in other *Holosporineae* of the genes for the R-bodies, involved in the killer trait conferred by ‘*Caedimonas*’ to its hosts, raises the question of whether similar mechanisms might be more pervasive among the *Holosporineae* and whether they might also have some implications in earlier evolutionary steps of this lineage [14]. This is even more so if we consider such phenomena as instances of addictive manipulation exerted by the bacteria on their hosts [43] and if we also take into account other cases among *Holosporineae* which could indicate addiction. These pertain ‘*Candidatus* Cytomitobacter spp.’ (*Holosporaceae*) and ‘*Candidatus* Bodonicaedibacter vickermanii’ (‘*Candidatus* Paracaedibacteraceae’), which, while devoid of R-bodies, could not be eliminated by antibiotics or cause the death of the host as well if removed, respectively [50, 57].

Finally, considering that multiple *Holosporineae* belonging to different families are pleomorphic, including the abovementioned *Holospora*/HLB, ‘*Caedimonas*’, and ‘*Candidatus* Hepatobacter’, as well as ‘*Candidatus* Finniella’ species [52, 75], it is interesting to wonder whether these are purely secondary lineage-specific adaptations or rely also on an ancestral morpho-functional ‘flexibility’ in the lifestyle of the ancestral *Holosporineae*.

## Final Remarks and Perspectives

Here, we provide an in-depth review of the literature on a broad, diverse, and ancient alphaproteobacterial lineage living in obligate association with eukaryotic hosts, mostly protists, aiming for a comprehensive dedicated resource for interested researchers. With the purpose of offering a common and stable nomenclature ground, we also propose a taxonomic revision based on phylogenetic and taxonomic considerations. Specifically, while recently these bacteria were commonly referred to either as an order (*Holosporales*) or a family (*Holosporaceae*) [1, 59], we move them as a suborder within the *Rhodospirillales*, namely the *Holosporineae*, keeping the internal taxonomic substructure of the *Holosporales *sensu Szokoli et al. [1].

We put forward that the knowledge on the diversity, host range, environmental, and geographical spread of the *Holosporineae*, though growing steadily through the years with continuous novel reports, is probably still an underestimate to date. Indeed, their most frequent hosts, protists, are still neglected in studies on associations with bacteria as compared to multicellular eukaryotes [41] and, despite being ecologically and geographically widespread [304], have little individual biomass, which may frequently hinder the detection of their associated bacteria, *Holosporineae* included, in environmental metagenomic screening studies.

Even in the most studied *Holosporineae Holospora* and ‘*Caedimonas*’, the interactions with the hosts, including mechanisms and reciprocal effects, are still insufficiently understood and almost entirely unknown in most of the other members of this lineage. This is quite unfortunate, not only for the sake of scientific and evolutionary curiosity on this neglected lineage, but also for several other relevant reasons. One of those is the still underappreciated, but well-plausible, impact that *Holosporineae* may have on aquatic ecosystems, based on the marked effects they have on certain widespread hosts, such as the competitive advantages conferred to the ‘killer’ paramecia by ‘*Caedimonas*’ [6]. Moreover, we underline the still not well-exploited convenience of using *Holosporineae* in comparative studies with other more renowned bacterial lineages sharing lifestyle features, such as the other professional symbionts [41]. This applies in particular to the *Rickettsiales* [40, 299], as these two lineages are independent instances of evolutionarily long-lasting associations with eukaryotes among the *Alphaproteobacteria*, thus offering a way to discern traits that are common, and thus more likely fundamental, from lineage-specific ones.

It is also worthwhile to consider the direct economical impact of those *Holosporineae* that affect farmed crustaceans [49, 291]. Additionally, we should not overlook that some *Holosporineae* are frequently associated with human pathogenic amoebas [11, 62, 137, 138, 149, 150, 152] and that DNA of others was found associated to humans [91, 93, 94, 163]. The latter findings may indicate a yet-to-be-confirmed direct association with human cells or the presence of undetected skin protists/fungi intracellularly hosting the bacteria. All these studies suggest a hypothetical role of the bacteria in the diseases [11], with potential analogies with the *Wolbachia* symbionts of filarial nematodes [305], and thus deserving further targeted investigations.

To sum up, accounting for evolutionary, ecological, economical, and possibly sanitary reasons, we highlight the need for future investigations to reveal the diversity of the *Holosporineae* and elucidate their functional interactions with eukaryote, which may be hopefully fostered and sustained by the recent and possible future increased availability of genomic sequences of these bacteria.

## Taxonomic Proposals

### Description of the Species ‘*Candidatus* Odyssella acanthamoebae’ comb. nov.

‘*Candidatus* Odyssella acanthamoebae’ (O.dys.sel’la. a.can.tha.moe’bae, N.L. fem. pl. n.). This corresponds to the description of ‘*Candidatus* Paracaedibacter acanthamoebae’ [11], with the following modifications. Phylogenetic position, family ‘*Candidatus* Paracaedibacteraceae’; intracellular symbiont of *Acanthamoeba* sp. UWC9 and other *Acanthamoeba* strains [11, 149–151].

### Emended Description of the Genus ‘*Candidatus* Paracaedibacter’ Horn et al. 1999

‘*Candidatus* Paracaedibacter’ (Pa.ra.cae.di.bac’ter, N.L. masc. s. n.). The genus contains only one described species, namely ‘*Candidatus* Paracaedibacter symbiosus’ [11]. Is the type genus of the family ‘*Candidatus* Paracaedibacteraceae’ [75].

### Emended Description of the Family ‘*Candidatus* Hepatincolaceae’ Szokoli et al. 2016

‘*Candidatus* Hepatincolaceae’ (He.pat.in.co.la’ce.ae, N.L. fem. pl. n.). The description of ‘*Candidatus* Hepatincolaceae’ [1] is emended as follows. The family currently contains three genera, ‘*Candidatus* Hepatincola’ [12], ‘*Candidatus* Tenuibacter’ [79], and ‘*Candidatus* Tardigradibacter’ [81], and is affiliated to the order *Rhodospirillales*. The type genus is ‘*Candidatus* Hepatincola’.

### Description of the Suborder *Holosporineae *subord. nov.

*Holosporineae* (Ho.lo.spo.ri’ne.ae, N. L. fem. n., *Holospora* type genus of the suborder; suff. -ineae ending to denote a suborder; N.L. fem. pl. n. *Holosporineae*, the suborder of the genus *Holospora*). The description is the same as that given previously for *Holosporales* [1], with some modifications. Defined by phylogenetic analyses based on SSU rRNA gene sequences and on concatenated conserved protein-coding ortholog genes. The suborder contains three families (*Holosporaceae*, ‘*Caedimonadaceae*’, and ‘*Candidatus* Paracaedibacteraceae’). The suborder *Holosporineae* is a member of the order *Rhodospirillales*.

## Data Availability

No datasets were generated or analysed during the current study.
